# Nutrients and Quality Aspects Characterizing *Ostrea edulis* Cultivated in Valli di Comacchio (Northern Italy) Across Different Seasons

**DOI:** 10.3390/molecules29235546

**Published:** 2024-11-24

**Authors:** Francesco Chiefa, Paola Tedeschi, Mirco Cescon, Valentina Costa, Elena Sarti, Manuel Salgado-Ramos, Noelia Pallarés, Natasha Damiana Spadafora, Leonardo Aguiari, Luisa Pasti

**Affiliations:** 1 Department of Chemical, Pharmaceutical and Agricultural Sciences, University of Ferrara, Via Luigi Borsari 46, 44121 Ferrara, Italy; francesco.chiefa@unife.it (F.C.); mirco.cescon@unife.it (M.C.); elena.sarti@unife.it (E.S.); 2 Department of Environmental and Prevention Sciences, University of Ferrara, Via Luigi Borsari 46, 44121 Ferrara, Italy; valentina.costa@unife.it (V.C.); damiana.spadafora@unife.it (N.D.S.); luisa.pasti@unife.it (L.P.); 3 Research Group in Innovative Technologies for Sustainable Food (ALISOST), Department of Preventive Medicine and Public Health, Food Science, Toxicology and Forensic Medicine, Faculty of Pharmacy, Universitat de València, Avda. Vicent Andrés Estellés, s/n, Burjassot, 46100 Valencia, Spain; manuel.salgado@uclm.es (M.S.-R.); noelia.pallares@uv.es (N.P.); 4 Naturedulis s.r.l., Piazzale Leo Scarpa 45, 44020 Ferrara, Italy; leonardo@naturedulis.com

**Keywords:** oyster, seasonal trend, nutritional quality, biochemical composition, food safety

## Abstract

The quality aspects of *Ostrea edulis* (*O. edulis*) cultured in Valli di Comacchio were examined across different seasons. Nutritional quality parameters, antioxidant activity, total carotenoids, and contaminants were determined in winter, summer, and autumn (December, June, and October). Seasonal variations in nutritional parameters were observed. In particular, in the winter samples, proteins, eicosapentaenoic acid, docosahexaenoic acid, threonine, tyrosine, valine and methionine, isoleucine, potassium, and carotenoids showed the highest values, whereas oleic acid, linolenic acid, lysine, and magnesium exhibited the maximum values in the summer. Finally, lipids, carbohydrates, histidine, and magnesium were present at high values in the autumn. The antioxidant activity values differed between the two methods used (DPPH and photochemiluminescence assays); however, the oysters collected in June presented lower antioxidant capacity. Contaminant levels were always below the imposed concentration limits; however, higher levels of toxic metals were detected in the winter, while polycyclic aromatic hydrocarbons were detected in the summer and autumn. It is important to monitor the nutritional and toxicological quality of *Ostrea edulis* throughout the cultivation seasons, not only to enhance knowledge of this species and improve its cultivation systems but also to determine the optimal period for human consumption from both nutritional and toxicological perspectives.

## 1. Introduction

In Europe, the conservation of the population of the European flat oyster, *Ostrea edulis* (Linnaeus 1758), constitutes the focus of ecological restoration efforts in order to profit from the ecosystem services of this biogenic reef-engineer species [1,2]. Furthermore, oyster is appreciated for its culinary, ecological, and medicinal value [3,4]. As a marine organism, it is part of ecosystems that serve as rich sources of novel bioactive compounds. These compounds have shown promising potential in fields such as drug discovery, providing new opportunities for developing treatments for diseases like cancer, infections, and inflammation. The unique physiological and biochemical adaptations of *Ostrea edulis (O. edulis)* to its marine environment make it particularly valuable in biotechnological and pharmaceutical research, offering innovative prospects for therapeutic applications and advancing our understanding of marine biodiversity [5,6].

However, throughout the 20th century, European stocks of *O. edulis* were severely depleted by overfishing, leading to numerous reseeding and restocking projects, mostly based on shellfish translocations from within Europe and from non-European areas [1,3,4,7]. These translocations are most likely responsible for the introduction and dispersal of parasites. In Europe, the late 1960s was characterized by marteliosis, followed by bonamiosis in 1979, which collectively led to a dramatic reduction in the stocks and beds of *O. edulis* throughout European waters [3,7,8,9]. The aquaculture of *O. edulis* has been greatly affected by these diseases, leading most oyster industries to shift away from cultivating this species. This shift has had obvious consequences, including moving toward the production of other shellfish species of commercial interest, such as Pacific oysters (*Magallana* or *Crassostrea gigas*) [3]. Fortunately, *O. edulis* is now at the core of many scientific projects and actions undertaken by governmental and non-governmental organizations for its aquaculture, restocking, and reintroduction all over European coasts. Indeed, since 2013, there has been a rapid increase in *O. edulis* restoration projects (there are now more than 30) in response to the severely degraded and depleted status of this species [4,10]. Nevertheless, knowledge of ecophysiological and environmental drivers is still limited for this species, limiting the development of successful breeding methods [3]. To the best of our knowledge, only a few recent studies on the seasonal changes in the biochemical and chemical composition of *O. edulis* [4,11,12,13] have been carried out, and no data are available on the organic contaminants of *O. edulis* cultured in Italy. Furthermore, no recent information is available regarding the biological performance of the European flat oyster, *O. edulis*, from a biochemical, nutritional, and contaminant-oriented point of view. Although *O. edulis* represents a valuable biological resource with a wide variety of bioactive compounds and functional properties, the concentration and quality of these molecules are often closely linked to the reproductive activity of marine organisms, which is influenced by environmental factors such as temperature and nutrient availability [11,14,15,16]. Carefully assessing the biochemical composition of this species over different seasons allows for the identification of optimal periods for human consumption tailored to specific nutritional needs. Additionally, seasonal toxicological monitoring can enhance the quality of bioactive molecules, ensuring safer and more effective use of marine resources for human nutrition and health.

In this paper, we present the findings of a study that evaluated the seasonal variations in several quality aspects of *O. edulis* cultivated in Valli di Comacchio (Emilia-Romagna, Italy). We focused on biochemical and nutritional characteristics, as well as safety concerns related to the presence of toxic metals and polycyclic aromatic hydrocarbons (PAHs). Oysters were analyzed in three different months: December, June, and October. The objective of this work is to assess the biochemical and toxicological aspects of *O. edulis* across different seasons. This research provides valuable insights into the European flat oyster, *O. edulis*, that can aid the production of this native species and encourage its reintroduction into the aquaculture industry.

## 2. Results and Discussion

### 2.1. Biochemical Analysis

The moisture, protein, total lipid, ash, and carbohydrate proportions are shown in Table 1. Proteins were the main component in the oysters harvested in December and June, while carbohydrates were the principal component in October.

During the sampling months, the lipid content reached a significant maximum value in October (*p* < 0.05) compared to December and June. This trend aligns with the studies on *O. edulis* by Abad et al. (1995), Ruiz et al. (1992), and Yildiz et al. (2011) [13,15,17], which reported that lipids reach a minimum level in winter and summer, corresponding to the initial phase of gametogenesis and the spawning phase, respectively. Subsequently, in the autumn, during the gonadal resting phase associated with a phytoplankton bloom, lipids accumulate in the tissues. The stored lipids are rapidly used up to overcome the stage of lowest food availability in late autumn. However, our findings differ from those of Pogoda et al. (2013) [4], who observed higher lipid levels in June than in October. This variation may be explained by the reproductive behavior of *O. edulis*, as it does not reproduce during the first year after settlement, leading to relatively constant lipid levels [4]. Furthermore, in our study, the trend of carbohydrates is similar to the change in lipids, suggesting that *O. edulis* utilizes both lipids and carbohydrates as energy sources during the same period. This observation is consistent with the studies of Abad et al. (1995) and Ruiz et al. (1992) [15,17]. Other researchers [4,18] have reported peaks in carbohydrate levels in *O. edulis* during the summer, followed by a decrease in autumn/winter. Nevertheless, the minor variations in the lipid and carbohydrate levels across the sampled months are likely influenced by the oysters’ stored energy, which they utilize during gametogenesis. Indeed, in a 1976 study, Bayne [19] stated that the stored energy that bivalves use for gametogenesis is common to both conservative and opportunistic species. In our study, *O. edulis* likely exhibited opportunistic behavior because there was sufficient food supply in Valli di Comacchio during the testing period, which helped prevent excessive variation in the percentages of lipids and carbohydrates.

Many researchers have reported that protein is the major structural material of gametes in some bivalves [20]. In Table 1, our results show that proteins are one of the main biochemical components of *O. edulis,* consistently exceeding 6% of the wet mass (WM). Moreover, there was a significant (*p* < 0.05) increase in protein content in December, probably because this period marks the beginning of gonadal development and, thus, an increase in protein synthesis [11,21].

Seasonal changes in the composition of fatty acids are shown in Table 2. Unsaturated fatty acids were predominant in *O. edulis* in December and June, although palmitic acid (C16:0) was the principal fatty acid (corresponding to about 28%) in December. In June, the levels of unsaturated fatty acids were significantly higher (*p* < 0.05) than those in December and October, as shown by high levels of palmitoleic acid (C16:1 n-7), oleic acid (C18:1 n-9), linoleic acid (C18:2 n-6)**,** linolenic acid (C18:3 n-3), and gondoic acid (C20:1 n-11). Particularly noteworthy is the high percentage of eicosapentaenoic acid (C20:5 n-3) observed in December.

**Figure 1:**
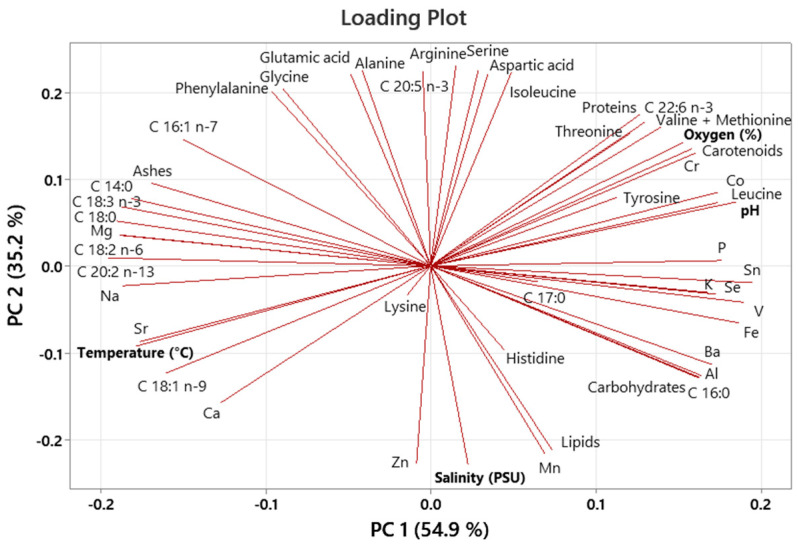
Loading plots of *Ostrea edulis* from the principal component analysis of the biochemical components (proximate composition, fatty acids, amino acids, and minerals) and environmental parameters (oxygen percentage, salinity, and temperature).

The quantity of fatty acids can be used as an indicator of the preferred diet. Indeed, assessments of fatty acids reflect bivalves’ food sources on a longer-term basis (weeks–months), as they measure nutrients assimilated in their tissues [18]. In our study, the fatty acids of oysters sampled in December were characteristic of dinoflagellate markers (C22:6 n-3, C16:1/C16:0 < 1, and C22:6 n-3/C20:5 n-3 > 1) [22,23]; in fact, according to environmental data reported by ARPAE (Emilia-Romagna Environmental Energy Prevention Agency) [24], during the winters of 2021 and 2022, the class Dinophyceae was the most present in Valli di Comacchio. The loading plot of the PCA (Figure 1) shows an inverse correlation between C22:6 n-3 (docosahexaenoic acid) percentage and temperature, likely due to the association of this fatty acid with the class Dinophyceae, which thrives during the colder seasons. In June, the fatty acids of *O. edulis* were characterized by C18:0 (C18:0, C18:1 n-9, C18:2 n-6, and C18:3 n-3) and C20:0 fatty acids (C20:2 n-13 and 16 and C20:5 n-3), reflecting diatoms and phytoplankton markers [25,26]. In particular, 18:2 n-6 could be derived from terrestrial plants, seagrasses, or some protozoa [27,28,29]. In fact, the PCA results indicate a positive correlation between C18 fatty acids and temperature, likely due to the composition of available food sources. In the autumn (October), the fatty acids of *O. edulis* were mainly characterized by palmitic acid (C16:0) and oleic acid (C18:1 n-9), indicating that the type of particles ingested was primarily composed of detritus [30,31]. It is likely that the oysters experienced periods in which the abundance of phytoplankton was too low to satisfy their energy demands. Typically, bivalves’ fatty acid compositions reflect the fatty acid compositions of their diets; however, bivalves have shown some ability to elongate or desaturate fatty acids [25].

**Figure 2:**
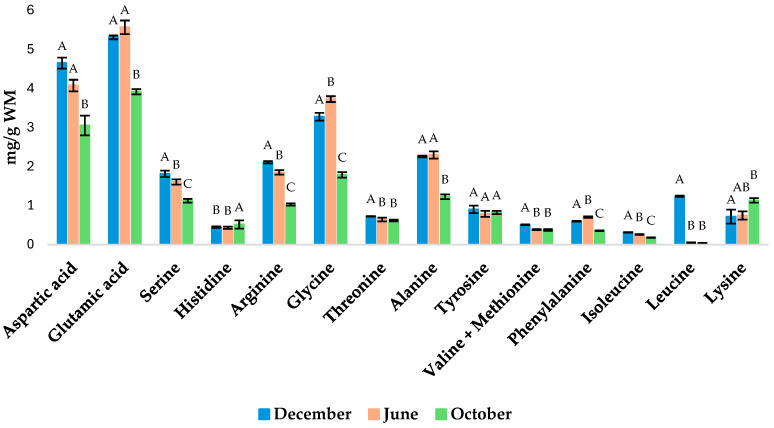
Results regarding the seasonal quantitative determination of total amino acids in *Ostrea edulis*. The results are expressed as means (*n* = 3) ± SD. Results from ANOVA show significant differences between the means. Means that share a common letter (A, B, C) are not significantly different from each other (Tukey’s test, *p* < 0.05).

The individual amino acid concentrations are reported in Figure 2. HPLC (high-performance liquid chromatography) conditions allowed us to separate and identify all the amino acids except for valine and methionine; these two were quantified together. The most abundant amino acids in *O. edulis* were aspartic and glutamic acid during the three sampling seasons, followed by glycine (Figure 2). Glutamic acid is one of the most flavorful amino acids, while glycine provides the most distinguished sweet taste in seafood [32]. All other amino acids were below about 0.4% WM.

Amino acids play a major role in taste as well as in other nutritional and biological aspects. Essential amino acids cannot be synthesized in the human body and must be obtained from daily food [33,34]. In *O. edulis*, the essential amino acids identified were comparable to those reported by Fu et al. (2024) for *Crassostrea gigas* [16]. However the levels of aspartic acid, serine, glutamic acid, glycine, alanine, and tyrosine values were lower in their study compared to ours. Furthermore, the seasonal variations in concentrations of aspartic acid, glutamic acid, arginine, glycine, alanine, leucine, and lysine were likely due to the amino acid composition of their diet. In fact, oysters fed with marine microalgae, such as *Phaeodactylum tricornutum*, *Platimonas viridis*, *Dunaliella tertiolecta*, and *Nefrochloris salina*, exhibited high levels of leucine and lysine [35]. Another study confirmed that the amino acid profiles of five less common marine organisms were highly dependent on food availability [36]. Indeed, the ARPAE report [24] confirms that the availability and composition of food in Valli di Comacchio change over the seasons. On the other hand, in the PCA loading plot (Figure 1), the weak relationships between some amino acids (phenylalanine, glycine, glutamic acid, alanine, arginine, serine, aspartic acid, and isoleucine) and oxygen percentage, salinity, temperature, and pH confirm the hypothesis that some amino acids are dependent on food availability rather than the abiotic parameters measured. Ultimately, these amino acids regulate key metabolic pathways, enhancing the health, survival, growth, development, lactation, and reproduction of the organisms, thereby increasing the nutritional value of *O. edulis* [37].

Bivalve mollusks are generally regarded as a good source of nutritionally important minerals. The mineral levels exhibited wide fluctuation over the seasons (Table 3). In particular, chromium, cobalt, potassium, phosphorus, selenium, and vanadium reached maximum levels in December; magnesium, sodium, and strontium reached maximum levels in June; and aluminum, barium, calcium, iron, magnesium, and zinc exhibited maximums in October. These fluctuations are likely related to changes in food supply, metal run-off linked to precipitation, and the reproductive cycle of mollusks [38,39]. The other metals exhibited approximately the same trend during the different seasons, with some significant differences (*p* < 0.05).

The values of aluminum, manganese, selenium, and potassium identified in our study are consistent with those reported by Erkan et al. in 2011 for *O. edulis* across different seasons [12]. However, the same study showed lower values of iron and calcium and higher values of zinc, cobalt, tin, chromium, sodium, magnesium, and potassium in different seasons compared to those shown here. In addition, Meloni et al. (2024) [40] reported mineral values in *Crassostrea gigas* that were comparable to those in our study for aluminum, iron, manganese, vanadium, and zinc; however, noted lower levels of cobalt and chromium and higher levels of selenium and tin. Nevertheless, according to the Scientific Committee on Food [41], the calcium, iron, magnesium, manganese, selenium, and zinc values of *O. edulis* are below the reference tolerable upper intakes for adults (considering the edible mass of a single individual).

In the end, the seasonal difference in the biochemical composition of *O. edulis* is closely related to its reproductive activity, which is affected by many environmental factors, primarily temperature and food supply [11,15,16].

Figure 1 illustrates the PCA (principal component analysis) results for *O. edulis*, with the first two components explaining 90.1% of the variance. The overall data on the biochemical and environmental parameters measured for *O. edulis* during the testing period were pooled and visualized using a PCA loading plot, highlighting a positive correlation between C18 fatty acids (C18:3 n-3, C18:0, C18:2 n-6, and C18:1 n-9) and water temperature, while some amino acids (phenylalanine, glycine, glutamic acid, alanine, arginine, serine, aspartic acid, and isoleucine) and the C20:5 n-3 (eicosapentaenoic acid) concentration show weak relationships with the measured environment parameters. Most mineral values, carotenoids, some amino acids (valine + methionine, leucine, tyrosine, and threonine), and proteins correlate to the percentage of oxygen and pH of water. Notably, the fatty acid C 22:6 n-3 (DHA) is inversely correlated with the water temperature.

### 2.2. Antioxidant Activity and Carotenoids

As shown in Table 4 the highest values for the photochemiluminescence (PCL) assay and total carotenoids were in December, while the maximum for the DPPH (2,2-diphenyl-1-picrylhydrazyl) assay was reached in October. All the values showed significant differences (*p* < 0.05) in the three seasons.

**Figure 3:**
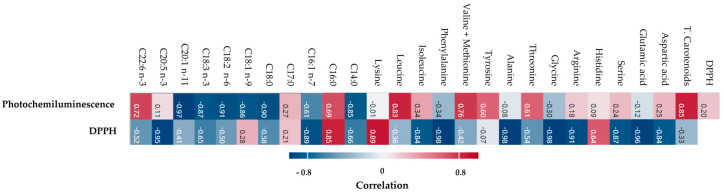
Pearson’s correlation between antioxidant capacity assays (photochemiluminescence and DPPH assay) and fatty acids, amino acids, and total carotenoids.

The heat map shows the correlation between fatty acids, amino acids, and total carotenoids and the antioxidant capacity assays (Figure 3). The photochemiluminescence assay values are higher in December (Table 4) than in other months and exhibit a correlation with docosapentaenoic acid (C22:6 n-3), leucine, valine + methionine, tyrosine, threonine, and carotenoid values (Figure 3). In fact, in December, these molecules presented higher values than in other months (see Table 2 and Figure 2). In October, the maximum value for the DPPH assay (IC_50_ = 60.52 ± 1.30), compared to the values in December and June (IC_50_ = 76.78 ± 1.22 and 104.78 ± 1.59, respectively), was reached and showed a correlation with palmitic acid (C16:0), lysine, and histidine values (Figure 3), which in the same month are most present in *O. edulis* (see Table 2 and Figure 2). The variability in the *O. edulis* antioxidant activity results is due to the methods used. None of the methods are ideal because each of them allows the measurement of different groups of antioxidants [42]. The DPPH molecule has little similarity with the highly reactive and transient peroxyl radicals. Furthermore, many antioxidants that may react quickly with peroxyl radicals in vivo may react slowly or even be inert to DPPH [43]. The analysis of photochemiluminescence, however, provides much more information on the biologically active components of *O. edulis*, showing a highly significant correlation with them (see Figure 3). However, the two methods present a low positive correlation (r = 0.20) between them (Figure 3).

Nevertheless, as evidenced by numerous studies, many substances, such as peptides, phenolic compounds, amino acids, lipids, and carotenoids, play important roles in the antioxidant activity of mollusks [44,45,46,47].

The total carotenoid content of *O. edulis* cultivated in Valli di Comacchio ranged from 1.850 µg/g in the summer to 7.759 µg/g in the winter (Table 4). These values are slightly lower than those reported in studies on *O. edulis* cultivated in the lagoon of Venice conducted by Czeczuga in 1979 [48]. Indeed, the total carotenoid content varies in individuals of the same species collected from different sites [48], but it is also dependent on factors such as sexual maturity, seasonal variation, and the source of dietary algae [49]. However, the carotenoids measured in the extracts used for antioxidant assays (photochemiluminescence and DPPH assay) contributed more positively to the photochemiluminescence assay (r = 0.85) than to the DPPH assay.

### 2.3. Contaminant Analysis

Bivalve mollusks are generally regarded as a good source of nutritionally important minerals; however, they are also capable of accumulating toxic metals present in the aquatic environment in their tissues. Table 5 reports the levels of arsenic, cadmium, lead, and mercury detected in the edible part of *O. edulis* cultivated in Valli di Comacchio. In December, these toxic metals had significantly (*p* < 0.05) higher values compared to those in June and October, except for cadmium. The high arsenic, lead, and mercury levels shown in winter are likely due to climatic factors such as precipitation [40,50].

The concentrations detected for cadmium, lead, and mercury were lower than the limits set by the European Commission (Table 5) [51], indicating no health concerns about trace element levels. The levels of arsenic in shellfish are not regulated by the European community [12]. The arsenic values identified in this study are comparable with those reported in studies on mussels from the Italian coast of the Adriatic Sea [52,53] and lower than those from other parts of the Adriatic Sea [54]. Nevertheless, arsenic can be present in sediments, resulting in bioaccumulation within bivalves. In fact, according to Amorosi and Sammartino (Amorosi and Sammartino, 2024), arsenic in the river Po may be derived from natural origins via Holocene peat bogs. Additionally, as noted by Sörös et al. in 2003 and Bergés-Tiznado et al. in 2013 [55,56], inorganic toxic arsenic present in seawater can be transformed in oyster tissues through a process called biodetoxification, resulting in over 90% conversion to organic, non-toxic forms. Subsequently, these organic arsenic compounds are further metabolized into dimethylarsinate, which is the primary metabolite excreted in urine in humans [57].

The concentrations of 15 polycyclic aromatic hydrocarbons (PAHs) were determined from the whole oyster tissues (Table 6). The individual polycyclic aromatic hydrocarbon concentrations fluctuated during the seasons, with the highest values mostly found in October.

The concentration of benzo(a)pyrene in *O. edulis* cultivated in Valli di Comacchio during the different seasons was below the limits set by the European Commission (5.0 µg/Kg WM) [58]. The combined levels of benzo(a)pyrene, benzo(a)anthracene, benzo(b)fluoranthene, and chrysene were 0.34, 0.74, and 1.78 ng/Kg WM in December, June, and October, respectively, all below the European Commission limits (30 µg/Kg WM) [51].

The polycyclic aromatic hydrocarbon concentrations of the oysters cultivated in Valli di Comacchio are comparable to those from other Adriatic Sea areas and lower than those in mussels cultivated in Taranto, Trieste, and the Gulf of Naples, areas close to significant anthropic impacts [59,60,61]. The average mussel dry mass is about 20%, and a similar range in mussels was reported for mussels off the Libyan coast in the Southern Mediterranean Sea (16.8–42.8 ng/g DM) [62]. Although the investigated area (Valli di Comacchio) is affected by intense maritime traffic and oil terminals, concentrations comparable to other areas of the Mediterranean were found.

In conclusion, *O. edulis* has proven to be not only an ecological asset but also a valuable nutritional and functional resource. It contains bioactive molecules important for human health, including omega-3 fatty acids, essential amino acids, and minerals. In particular, proteins, omega-3 fatty acids, essential amino acids, and some minerals exhibited higher values during the winter. However, the presence and high concentrations of these molecules are highly influenced by environmental and biological factors such as temperature, food availability, gametogenesis, and the spawning phase, which vary across different seasons. Furthermore, to better understand the antioxidant capacity of oysters, it is advisable to use various antioxidant assays, as doing so allows for the evaluation of the antioxidant power of different molecules, which may vary over different seasons.

Our study, conducted in a brackish basin in Valli di Comacchio, shows that this area can be considered a suitable and promising site for *O. edulis* farming. The results demonstrate that the farming of *O. edulis* in Valli di Comacchio is interesting not only from a nutritional perspective but also from a food safety standpoint. Today, food safety is a priority for governments, politicians, the food industry, and researchers worldwide, and the farming of *O. edulis* in Valli di Comacchio has proven to be in line with the concept of food safety.

## 3. Materials and Methods

### 3.1. Study Area and Experimental Design

The European flat oyster (*O. edulis*) was cultivated in Valli di Comacchio, Emilia-Romagna, Italy, in the Northern Adriatic Sea. The environment parameters of water (oxygen percentage, salinity, temperature, and pH) used for PCA are shown in the ARPAE’s monthly reports [63,64,65].

Juvenile oysters were obtained from Naturedulis S.r.l. hatcheries located in Goro (Ferrara, Emilia-Romagna, Italy). *O. edulis* spat (mass weight: 2.5 ± 0.4 g; shell length: 31.3 ± 2.8 mm upon attachment) were placed into floating cages of the FlipFarm™ system (Middle Renwick, Blenheim, New Zealand) stocked at 100 individuals/cage. This study was conducted over one complete growing season from December 2021 to October 2022. During the trial, about 1 kg of *O. edulis* (25 individuals) specimens were collected for each season (December, June, and October) from 2021 to 2022. The shells were opened carefully, and the entire soft body (the edible part) was removed from the shell. The biochemical composition (moisture, protein, ash, lipid amount, lipid profile, total amino acids, and metals), antioxidant activity, carotenoids, and contaminant components (toxic metals and polycyclic aromatic hydrocarbons) in the soft body were analyzed during the three seasons.

### 3.2. Biochemical Analysis

Moisture. *O. edulis* samples with a total weight of about 1 kg (corresponding to 25 individuals) were carefully opened and blended with a grindermix (Reusch grindermix 200, Verder Scientific S.r.l., Bergamo, Italy). The samples were divided into 10 glass beakers and freeze-dried at −60 °C and 0.090 mbar (M. CHRIST, Alpha 1-2 LDplus, Osterode am Harz, Germany) for 48 h to attain a constant weight. The dry matter was used to quantify proteins, ashes, lipids, amino acids, and minerals, and the moisture percentage was utilized to calculate the oysters’ nutritional value in reference to fresh weight.

Proteins. Proteins (total nitrogen compounds) were determined in 0.6 g of freeze-dried samples according to the Kjeldahl method [66]. The protein content was determined by means of a conversion factor equal to 6.25 and expressed as a percentage of wet mass (WM).

Ashes. Total mineral content (ashes) was determined in 1 g of freeze-dried sample in a muffle furnace at 570 °C (Z1200, Zetalab, Padova, Italy) overnight [67]. Total ashes are expressed as a percentage of wet mass (WM).

Lipid Extraction. About 1.5 g of freeze-dried product was put in a thimble connected to a Soxhlet extraction unit (Velp Scientifica, Usmate, Milan, Italy). Extraction solvent: 50 mL of diethyl ether (Carlo Erba Reagents S.r.l, Milan, Italy). Extraction steps: 30 min with a thimble immersed in a boiling solvent, 30 min of reflux washing, and 10 min of solvent recovery [68]. Total lipids are expressed as a percentage of wet mass (WM).

Fatty acid composition. After extraction, fats were dissolved in 3 mL of hexane (Carlo Erba Reagents S.r.l, Milan, Italy) and transesterified with 1.5 mL of 5% sodium hydroxide in methanol (Carlo Erba Reagents S.r.l, Milan, Italy) to obtain free fatty acid methyl esters. A volume of 1 μL was injected into a gas chromatography–mass spectrometer (Varian 3900 gas-chromatograph, Varian, Palo Alto, CA, USA) coupled to a Varian Saturn 2100 MS/MS ion trap mass spectrometer. Separation was achieved with a capillary column Zebron ZB-WAX (60 m, 0.25 mm i.d., 25 μm film thickness, Phenomenex, Milan, Italy) supplied with helium carrier gas at 1 mL/min constant flow. The injector temperature was 260 °C, and the oven temperature program was as follows: start at 100 °C for 2 min, raise the temperature to 200 °C at 10 °C/min, and then hold for 58 min. The MS acquisitions were performed via electron ionization (EI) in full-scan mode with a mass range of *m*/*z* 40–650, and the collected data were evaluated using the NIST MS library [68].

Total amino acids. About 0.2 g of freeze-dried samples were weighed out and mixed with 10 mL of HCl 6M (37%, Superpure, Carlo Erba Reagents, Milan, Italy) into a tetrafluoro-methoxyl vessel and hydrolyzed in a microwave digestion system (Ethos Easy Advanced Microwave Digestion System, equipped with a SK15 rotor, Milestone S.r.l., Sorisole, Italy), according to the conditions reported by Wang et al. in 2022 [69]. Subsequently, hydrolyzed samples were filtered (Whatman 589/2), dried under a nitrogen flow in a bath at 50 °C, and finally resuspended in 0.1 M HCl (Merck, Darmstadt, Germany). The detection and quantification of amino acids were performed with a high-performance liquid chromatograph coupled with a fluorescence detector (Agilent Technologies 1260 Infinity, Santa Clara, CA, USA). The chromatographic conditions employed were in accordance with a modified version of the method described by Agilent methods [70]. Mobile phase A consisted of 50 mM of Na_2_HPO_4_ (Carlo Erba Reagents, Milan, Italy) in ultrapure water (MilliQ^®^, Merck KGaA, Darmstadt, Germany), adjusted to pH 7.5, while mobile phase B was acetonitrile/methanol/ultrapure water, 45:45:10 vol.% (VWR International Srl, Milan, Italy; Merck, Darmstadt, Germany). Briefly, 14 µL of hydrolyzed samples (diluted 1:20) or a standard amino acid mixture was added to 280 µL of Borate Buffer (0.1 M, pH 10.2) (Merck, Darmstadt, Germany), 14 µL of internal standard, and 140 µL of OPA (*o*-Phthaldialdehyde), 1 mg/mL (P0532, Merck, Darmstadt, Germany), as derivatization agents and brought up to 1 mL with water. As an internal standard, we used tryptophan (T0254, Merck, Darmstadt, Germany) because tryptophan and cysteine were destroyed during acid hydrolysis [71,72]. A total of 20 µL of each sample was injected into a Pursuit XRs 5 C18 150 mm × 4.6 mm column at 20 °C, with detection at λ_exicetement_ = 230 nm and λ_emission_ = 450 nm. The separation was performed at a flow rate of 0.7 mL/min, employing a solvent gradient (vol.%) as follows: 0 min for 2% mobile phase B, 2.5 min for 2% mobile phase B, 40 min for 60% mobile phase B, 45 min for 100% mobile phase B, and 50 min for 100% mobile phase B, pre-equilibrated under initial conditions for 15 min. Appropriate amounts of a standard amino acid solution, 2.5 µmol/mL (AAS18, Merck, Darmstadt, Germany), were used to prepare stock standard solutions ranging from 25 to 700 pmol/µL in triplicate. The calibration curves of each amino acid were obtained by plotting the peak area against concentration (R^2^ = 0.9935–0.9998). The amino acids analyzed were aspartic acid (15.56 min), glutamic acid (18.39 min), serine (24.84 min), histidine (25.19 min), arginine (26.88 min), glycine (28.23 min), threonine (28.47 min), alanine (31.42 min), tyrosine (32.07 min), valine + methionine (39.00 min), tryptophan (39.57 min), phenylalanine (41.15 min), isoleucine (42.66 min), leucine (43.48 min), and lysine (45.82 min). Amino acids are expressed as mg/g of wet mass (WM).

Metals. An Ethos Easy Advanced Microwave Digestion System equipped with an SK15 rotor (Milestone S.r.l., Sorisole, Italy) was employed for mineralization. About 0.4 g of each freeze-dried sample was weighed in a tetrafluoro-methoxyl vessel and supplemented with 9 mL of HNO_3_ (69%, Suprapur^®^, Merck, Darmstadt, Germany) and 1 mL of H_2_O_2_ (30%, Carlo Erba Reagents, Milan, Italy). The microwave program was 15 min at 200 °C with a 15-min holding time at a maximum power of 1800 W. Subsequently, mineralized samples were filtered (ashless filters, Whatman 589/2) into a 50 mL volumetric flask and filled with ultrapure water (MilliQ^®^, Merck KGaA, Darmstadt, Germany) to the mark.

An 8800 Inductively Coupled Plasma Triple Quadrupole Mass Spectrometer (ICP-MS, Agilent Technologies Inc., Santa Clara, CA, USA) was employed to quantify the following trace elements: arsenic, barium, cadmium, cobalt, chromium, iron, manganese, lead, tin, strontium, and vanadium. The ICP-MS was equipped with a Micro-Mist glass concentric nebulizer, a Peltier cooled double-pass Scott-type spray chamber, and Ni cones; the instruments’ acquisition parameters were 1550 W of RF power, an 8.0 mm sampling depth, 15 L/min of plasma gas, and 1.03 L/min of carrier gas, with the spray chamber temperature set to 2 °C; the isotopes measured were ^75^As, ^137^Ba, ^111^Cd, ^59^Co, ^52^Cr, ^56^Fe, ^55^Mn, ^208^Pb, ^118^Sn, ^88^Sr, and ^51^V, and the signals were collected using a single-quad scan in No Gas mode, He mode, and He-He mode with 0, 4.5, and 10 mL/min flows of collision cells, respectively. The integration time was 0.1s for each mass value, and data acquisition was fixed at 3 replicates and 100 sweeps for replicates. The samples were diluted by at least a 1:10 ratio with 1% HNO_3_ and 0.5% HCl (37%, Superpure, Carlo Erba Reagents, Milan, Italy) in ultrapure water. A multielement standard solution for ICP (Merck, Darmstadt, Germany) was used to prepare the calibration curves. 

An *Optima 3100 XL* inductively coupled plasma optical emission spectrometer (ICP-OES, Perkin-Elmer Inc., Shelton, CN, USA) was employed to quantify the following elements: aluminum (308.215 nm), calcium (315.887 nm), magnesium (279.077 nm), phosphorus (214.914 nm), selenium (196.026 nm), and zinc (213.857 nm), reported with analytical lines for quantitative determination. The ICP-OES was equipped with an axial torch, a segmented array charge-coupled device (SCD) detector, and a Babington-type nebulizer with a cyclonic spray chamber for sample introduction; the work conditions of plasma were an RF power of 1.40 kW, a 15 L/min flow rate, and 0.5 L/min for auxiliary gas; the flow rate of the nebulizer gas was 0.65 L/min. Multielement and P standard solutions applied at 1000 mg/L (Carlo Erba Reagents S.r.l., Milan, Italy) were used to prepare the calibration curves [73].

An *AAnalyst 800* atomic adsorption spectrometer (AAS, Perkin-Elmer Inc., Shelton, CN, USA) was employed to quantify sodium and potassium in emission at 766.5 nm and 589.0 nm, respectively. The AAS’s working conditions were air flow at 17.0 L/min, acetylene flow at 2.0 mL/min, and an integration time of 3 s for 3 replicates. Sodium and potassium standard solutions (100 mg/L) (Merck, Darmstadt, Germany) were used to prepare the calibration curves. Metals are expressed as mg/Kg of wet mass (WM) [73].

Biochemical compositions were analyzed in triplicate.

### 3.3. Antioxidant Activity and Carotenoids

The freeze-dried *O. edulis* samples were subjected to double extraction with an ethanol/water mixture (3:1 vol.%). About 0.5 g of samples were added to 5 mL of an ethanol/water mixture and placed under stirring conditions in the dark for 30 min. Subsequently, the samples were centrifuged at 2500× *g* for 10 min, and the supernatant was recovered; a second extraction was then carried out. The samples were then stored at −25 °C; subsequently, antioxidant capacity and carotenoid content were measured.

The antioxidant capacity of the *O. edulis* extracts was tested using a DPPH scavenging activity assay and a photochemiluminescence assay (Photochem^®^).

DPPH (2,2-diphenyl-1-picrylhydrazyl) assay. This assay was executed according to the method reported by Tedeschi et al. in 2023 [74]. Trolox (6-hydroxy-2,5,7,8-tetramethylchroman-2-carboxylic acid), 0.05–1 mM in methanol, was used to prepare a calibration curve. A deep-purple solution of DPPH (0.06 mM) was prepared in methanol, and the absorbance was measured at 515 nm using a UV-6300PC Double Beam Spectrophotometer (VWR International Srl, Milan, Italy).

Aliquots of 50 µL of *O. edulis* extracts at different concentrations were added to 1450 µL of the DPPH methanol solution; the mixture was stirred vigorously and kept for 15 min in the dark at room temperature. A decrease in spectrophotometric absorbance with the color changing toward yellow was registered. The antioxidant activity was calculated according to the percentage of inhibition of the DPPH radical:

$$Inhibition\;\left(\%\right)=\frac{\left(Control\;Abs\;\left(t=0\;min\right)\;-\;Sample\;Abs\;(t=15\;min)\right)}{Control\;Abs\;(t=0\;min)}\times 100$$

where Control Abs is the absorbance of the control (DPPH), and sample Abs is the absorbance of the standard or the sample. The calibration curve (R^2^ = 0.9914) was expressed as mM of Trolox equivalents.

The DPPH value was expressed in Trolox equivalents (µmol/g) using the following formula: IC_50_ Trolox (µmol/L)/IC_50_ extract (g/L) [75].

Photochemiluminescence (PCL) assay. This assay, based on the methodology reported by Maietti et al. in 2017 [76], was used to measure the antioxidant activity of the *O. edulis* extracts via a Photochem^®^ apparatus (Analytic Jena, Jena, Germany) against superoxide anion radicals generated from luminol. In particular, the antioxidant activity of the extracts was assessed by means of an ACL (antioxidant capacity of lipid substances) kit (Analytic Jena, Jena, Germany). For the ACL assay, 2.3 mL of reagent 1 (a solvent and dilution reagent, MeOH), 0.2 mL of reagent 2 (a buffer solution), 25 µL of reagent 3 (a photosensitizer, namely, luminol, 1 mmol/L), and 10 µL of standard or sample solution were mixed and measured. Luminol is used as a photosensitizer, showing activity when exposed to UV light at a maximum of 351 nm, and a detection substance for free radicals. Trolox is used for the standard calibration curve from 0.25 to 2 nM. In the PCL-ACL assay, the photochemical generation of free radicals is combined with sensitive detection realized using chemiluminescence. In the ACL studies, the kinetic light emission curve was monitored for 3 min and expressed as (µmol Trolox equivalents/g). The areas under the curves were calculated by using PCL soft control and analysis software. Trolox or antioxidants from the samples reduce the magnitude of the PCL signal and hence the area calculated from the integral. The observed inhibition of the signal was plotted against the concentration of Trolox added to the assay medium. The concentration of the added sample was such that the generated luminescence during the 3 min sampling interval fell within the limits of the standard curve.

Total carotenoids. This assay was carried out using UV-Vis spectroscopy via a UV-6300PC Double Beam Spectrophotometer (VWR International Srl, Milan, Italy), following the protocol described by Braniša et al. in 2014 [77]. The absorbance maxima of 1.5 mL of each extract were read at 663.6 nm for chlorophyll a, 646.6 nm for chlorophyll b, and 470.0 nm for carotenoids. The concentrations for carotenoids were calculated by following the equation in the study cited above. The results are expressed as µg/g of wet mass (WM).

The antioxidant activity and total carotenoids were analyzed in triplicate.

### 3.4. Contaminants Analysis

Toxic metals. Arsenic, cadmium, and lead were analyzed, as noted in a paragraph in Section 3.2 Biochemical Analysis.

An SMS 100 Mercury Analysis System (Perkin-Elmer Inc., Shelton, CN, USA) was employed for total mercury determination according to EPA method 7473 [78] through thermal decomposition; then, amalgamation on a gold trap, thermal desorption, and vapor analyses via atomic absorption spectroscopy according to EPA method 7473 were conducted [78]. A total of 0.5 g of each sample was weighed in a sample boat and inserted into the instrument for analysis, and the absorbance was measured at 253.7 nm as a function of mercury concentration.

Polycyclic aromatic hydrocarbons (PAHs). About 1 g of the freeze-dried sample was extracted with a 20 mL mixture of hexane/dichloromethane, 40:60 %vol. (Merck, Darmstadt, Germany), and 8 g of Florisil solid-phase extraction (Carlo Erba Reagents, Milan, Italy) for the purification of lipids. Afterward, the mixture was sonicated for 10 min and centrifuged for 5 min at 5000× *g*. The extraction was repeated two times. The extract was evaporated at room temperature under a nitrogen atmosphere until reaching a volume of 3–5 mL. Subsequently, 20 mL of KOH 0.5M (Carlo Erba Reagents, Milan, Italy) in methanol (Merck, Darmstadt, Germany) was added to the extract, and it was sonicated for 30 min until reaching 40 °C. Subsequently, the extraction was repeated three times with a hexane/dichloromethane mixture (80:20 %vol.). The extract was separated and evaporated at room temperature under a nitrogen atmosphere until reaching a volume of 1–2 mL, supplemented with acetonitrile (VWR International Srl, Milan, Italy), and concentrated until reaching a volume of 1 mL [79,80]. A total of 20 µL of the extract was injected into a high-performance liquid chromatography (HPLC) coupled with a fluorescence (FLD) detector (Agilent Technologies 1260 Infinity, Santa Clara, CA, USA). The chromatographic conditions employed were in accordance with EPA method 8310 [81]. Mobile phase A consisted of water (MilliQ^®^, Merck KGaA, Darmstadt, Germany), while mobile phase B was acetonitrile (VWR Chemicals). The samples were injected into an Agilent ZORBAX Eclipse PAH, 4.6 × 150 mm^2^, 5 µm column at 25 °C, with detection at λ_exicetement_ = 270 nm and λ_emission_= 330 nm (FLD A), 250–370 nm (FLD B), 330–430 nm (FLD C), 270–390 nm (FLD D), 290–430 nm (FLD E), and 260–500 nm (FLD F). The separation was performed at a flow rate of 1.5 mL/min, employing a solvent gradient (vol.%) as follows: 0 min of 40% B and 20 min of 95% B, re-equilibrated under initial conditions in 15 min. Appropriate amounts of standard polycyclic aromatic hydrocarbon solution (Merck, Darmstadt, Germany) were used to prepare stock standard solutions ranging from 1 to 50 ng/mL in triplicate. The calibration curves of each polycyclic aromatic hydrocarbon were obtained by plotting peak area against concentration (R^2^ = 0.9995–1). The polycyclic aromatic hydrocarbons analyzed were naphthalene (6.30 min), acenaphthene (8.79 min), fluorene (9.16 min), phenanthrene (10.27 min), anthracene (11.48 min), fluoranthene (12.55 min), pyrene (13.56 min), benzo(a)anthracene (16.63 min), chrysene (17.83 min), benzo(b)fluoranthene (19.93 min), benzo(k)fluoranthene (21.16 min), benzo(a)pyrene (22.09 min), dibenzo(a,h)anthracene (24.42 min), benzo(g,h,i)perylene (25.37 min), and indeno(1,2,3-cd)pyrene (27.13 min).

The contaminant analysis was conducted in triplicate.

### 3.5. Statistical Analysis

Statistical processing was performed using Minitab software version 20.4 (https://www.minitab.com/en-us/). A one-way analysis of variance (ANOVA) and Tukey’s test were used to examine the significant variation in biochemical composition, antioxidant capacity, carotenoids, and contaminant fingerprints between the different seasons (December, June, and October) in which *O. edulis* sampling was performed.

A heat map (Pearson correlation) was used to show the correlation between the antioxidant capacity assays and lipids, amino acids, and carotenoids (Pearson correlation, Minitab software version 20.4). The relationships between the DPPH assay and photochemiluminescence assay were evaluated using Minitab software version 20.4 (Pearson correlation).

A multivariate analysis of principal components (PCA) was used to examine the effects of environmental variables (oxygen percentage, salinity, temperature, and pH) on the biochemical composition of *O. edulis*, including with regard to proximate composition, lipid profile, amino acid profiles, and minerals (Minitab software, version 20.4).

*p* < 0.05 indicates that the difference is statistically significant at a 95% probability level, and the results are presented as means ± standard deviations.

## Figures and Tables

**Table 1:** Seasonal proximate composition of *Ostrea edulis*. The results, given as % of wet mass (WM), are expressed as means (*n* = 3) ± SD. Results from ANOVA showed significant differences between the means. In each row, the means that are not statistically different are indicated with the same letter (A, B, C) as a superscript (Tukey’s test, *p* < 0.05).

	December	June	October
Moisture	81.26 ± 0.06 ^B^	83.45 ± 0.07 ^A^	81.74 ± 0.57 ^B^
Lipids	1.11 ± 0.01 ^B^	0.99 ± 0.10 ^B^	1.66 ± 0.01 ^A^
Proteins	7.99 ± 0.02 ^A^	6.76 ± 0.08 ^B^	6.38 ± 0.05 ^C^
Ashes	2.01 ± 0.01 ^AB^	2.72 ± 0.32 ^A^	1.85 ± 0.02 ^B^
Carbohydrates *	7.66 ± 0.01 ^A^	6.14 ± 0.13 ^B^	8.36 ± 0.05 ^C^

* Calculated according to the difference between 100% and other components.

**Table 2:** Seasonal percentage of fatty acids in *Ostrea edulis.* The results are expressed as means (*n* = 3) ± SD. Results from ANOVA show significant differences between the means. In each row, the means that are not statistically different are indicated with the same letter (A, B, C) as a superscript (Tukey’s test, *p* < 0.05).

% Fatty Acids	December	June	October
C14:0	3.90 ± 0.37 ^B^	6.33 ± 0.24 ^A^	3.85 ± 0.21 ^B^
C16:0	28.82 ± 2.75 ^A^	6.28 ± 0.61 ^B^	41.67 ± 2.66 ^C^
C16:1 n-7	4.07 ± 0.19 ^A^	5.33 ± 0.19 ^B^	3.35 ± 0.14 ^C^
C17:0	2.32 ± 0.21 ^A^	1.87 ± 1.05 ^A^	2.44 ± 0.13 ^A^
C18:0	7.95 ± 0.44 ^B^	15.09 ± 0.75 ^A^	8.57 ± 0.16 ^B^
C18:1 n-9	7.26 ± 0.93 ^B^	13.06 ± 0.10 ^A^	12.59 ± 0.42 ^A^
C18:2 n-6	2.87 ± 1.04 ^B^	7.42 ± 0.06 ^A^	3.70 ± 0.12 ^B^
C18:3 n-3	4.82 ± 0.12 ^B^	13.13 ± 0.37 ^A^	4.63 ± 0.18 ^B^
C20:1 n-11	0.63 ± 0.01 ^A^	8.12 ± 0.46 ^B^	2.64 ± 0.21 ^C^
C20:5 n-3	14.11 ± 0.59 ^A^	13.67 ± 0.36 ^A^	9.48 ± 1.02 ^B^
C22:6 n-3	20.97 ± 1.34 ^A^	9.70 ± 0.97 ^B^	7.07 ± 1.58 ^B^
Saturated	43.98 ± 2.38 ^A^	29.57 ± 2.18 ^B^	56.54 ± 2.58 ^C^
Unsaturated	56.02 ± 2.38 ^A^	70.43 ± 2.18 ^B^	43.46 ± 2.58 ^C^
Monounsaturated (MUFA)	12.26 ± 1.15 ^A^	26.51 ± 0.54 ^B^	18.59 ± 0.07 ^C^
Polyunsaturated (PUFA)	43.76 ± 1.23 ^A^	43.92 ± 1.64 ^A^	24.88 ± 2.65 ^C^

**Table 3:** Mineral composition (mg/Kg WM) of *Ostrea edulis* in different seasons. The results are expressed as means (*n* = 3) ± SDs. Results from ANOVA show significant differences between the means. In each row, the means that are not statistically different are indicated with the same letter (A, B, C) as a superscript (Tukey’s test, *p* < 0.05).

Metals (mg/Kg WM)	December	June	October	Tolerable Upper Intake Level
Al	61.21 ± 2.89 ^A^	21.94 ± 0.41 ^B^	80.10 ± 1.64 ^C^	n/a
Ba	0.29 ± 0.01 ^A^	0.15 ± 0.01 ^B^	0.33 ± 0.01 ^C^	n/a
Ca	485.33 ± 37.22 ^B^	940.39 ± 0.01 ^A^	1032.47 ± 215.29 ^A^	2500 mg/day
Cr	3.21 ± 0.15 ^B^	0.26 ± 0.04 ^A^	0.41 ± 0.03 ^A^	n/a
Co	0.09 ± 0.01 ^B^	0.05 ± 0.01^A^	0.06 ± 0.01 ^A^	n/a
Fe	90.27 ± 5.81 ^A^	40.73 ± 0.59 ^B^	91.21 ± 0.69 ^A^	40 mg/day
K	2209.98 ± 125.60 ^A^	1980.32 ± 17.14 ^B^	2172.61 ± 13.03 ^A^	n/a
Mg	541.87 ± 26.25 ^B^	685.72 ± 3.44 ^A^	562.41 ± 6.58 ^B^	250 mg/day
Mn	3.86 ± 0.16 ^A^	3.30 ± 0.02 ^B^	6.86 ± 0.06 ^C^	8 mg/day
Na	1142.68 ± 65.43 ^A^	1623.70 ± 18.86 ^B^	1301.51 ± 13.23 ^C^	n/a
P	1140.50 ± 0.00 ^A^	1016.70 ± 4.24 ^B^	1098.36 ± 8.43 ^A^	n/a
Se	1.09 ± 0.06 ^A^	0.96 ± 0.01 ^B^	1.04 ± 0.05 ^AB^	255 µg/day
Sn	<0.002 ^A^	<0.002 ^A^	<0.002 ^A^	n/a
Sr	4.06 ± 0.14 ^B^	5.99 ± 0.36 ^A^	5.41 ± 0.45 ^A^	n/a
V	0.15 ± 0.01 ^A^	0.05 ± 0.01 ^B^	0.14 ± 0.01 ^A^	n/a
Zn	108.44 ± 3.79 ^A^	114.64 ± 0.75 ^B^	136.58 ± 0.84 ^C^	25 µg/day

n/a: no adequate data.

**Table 4:** DPPH assay results, photochemiluninescence assay results, and total carotenoids of *Ostrea edulis* collected in three different seasons. The results are expressed as means (*n* = 3) ± SD. Results from ANOVA show significant differences between the means. In each row, the means that are not statistically different are indicated with the same letter (A, B, C) as a superscript (Tukey’s test, *p* < 0.05).

	December	June	October
**DPPH assay** (µmol TROLOX eq./g)	6.38 ± 0.10 ^A^	4.68 ± 0.07 ^B^	8.10 ± 0.17 ^C^
**Photochemiluminescence (PCL) assay** (µmol TROLOX eq./g)	0.84 ± 0.02 ^A^	0.31 ± 0.03 ^B^	0.59 ± 0.04 ^C^
**Total carotenoids** (µg/g WM)	7.76 ± 0.02 ^A^	1.85 ± 0.07 ^B^	2.05 ± 0.03 ^C^

**Table 5:** Toxic metals (mg/Kg WM) in *Ostrea edulis* in different seasons. The results are expressed as means (*n* = 3) ± SD. Results from ANOVA show significant differences between the means. In each row, the means that are not statistically different are indicated with the same letter (A, B, C) as a superscript (Tukey’s test, *p* < 0.05).

Toxic Metals (mg/Kg WM)	December	June	October	Limits Imposed by the European Commission (mg/Kg WM)
As	4.23 ± 0.22 ^A^	2.62 ± 0.03 ^B^	2.85 ± 0.02 ^B^	n/a
Cd	0.20 ± 0.02 ^A^	0.26 ± 0.01 ^B^	0.21 ± 0.01 ^B^	1.0
Pb	0.09 ± 0.00 ^A^	0.08 ± 0.00 ^B^	0.09 ± 0.00 ^C^	1.5
Hg	0.03 ± 0.00 ^A^	0.02 ± 0.00 ^B^	0.02 ± 0.00 ^C^	0.5

n/a: no adequate data.

**Table 6:** Concentrations (ng/Kg WM) of polycyclic aromatic hydrocarbons in *Ostrea edulis* in different seasons. The results are expressed as means (*n* = 3) ± SD. Results from ANOVA show significant differences between the means. In each row, the means that are not statistically different are indicated with the same letter (A, B, C) as a superscript (Tukey’s test, *p* < 0.05).

PAH (ng/g WM)	December	June	October
Naphthalene	<0.01 ^A^	1.22 ± 0.47 ^B^	0.28 ± 0.11 ^A^
Acenaphthene	<0.01 ^B^	0.03 ± 0.01 ^A^	0.04 ± 0.00 ^A^
Fluorene	0.23 ± 0.04 ^A^	0.36 ± 0.04 ^A^	0.23 ± 0.06 ^A^
Phenanthrene	0.11 ± 0.07 ^A^	1.11 ± 0.08 ^B^	1.80 ± 0.19 ^C^
Anthracene	<0.2 ^A^	<0.2 ^A^	<0.2 ^A^
Fluoranthene	0.64 ± 0.11 ^A^	1.32 ± 0.04 ^AB^	2.09 ± 0.76 ^B^
Pyrene	0.70 ± 0.10 ^B^	1.37 ± 0.07 ^A^	1.26 ± 0.09 ^A^
Benzo(a)anthracene	0.03 ± 0.01 ^A^	0.01 ± 0.00 ^A^	0.03 ± 0.01 ^A^
Chrysene	0.11 ± 0.01 ^B^	0.14 ± 0.01 ^B^	0.91 ± 0.09 ^A^
Benzo(b)fluoranthene	0.06 ± 0.05 ^A^	0.45 ± 0.03 ^B^	0.70 ± 0.08 ^C^
Benzo(k)fluoranthene	<0.14 ^A^	<0.14 ^A^	0.27 ± 0.03 ^B^
Benzo(a)pyrene	<0.14 ^B^	<0.14 ^A^	<0.14 ^A^
Dibenzo(a,h)anthracene	0.10 ± 0.01 ^A^	0.04 ± 0.02 ^B^	0.05 ± 0.00 ^AB^
Benzo(g,h,i)perylene ene	0.05 ± 0.01 ^A^	0.09 ± 0.00 ^B^	0.07 ± 0.01 ^AB^
Indeno(1,2,3-cd)pyrene	<0.04 ^A^	0.07 ± 0.02 ^B^	0.19 ± 0.00 ^C^
∑PAHs	2.33 ± 0.30 ^A^	5.06 ± 1.77 ^AB^	7.55 ± 1.39 ^B^

## Data Availability

Data are contained within the article.
